# Integrated Network Pharmacology and GC-MS–Based Metabolomics to Investigate the Effect of Xiang-Su Volatile Oil Against Menopausal Depression

**DOI:** 10.3389/fphar.2021.765638

**Published:** 2021-12-02

**Authors:** Yao Li, Xinyi Yang, Shanshan Chen, Lei Wu, Jinyong Zhou, Keke Jia, Wenzheng Ju

**Affiliations:** ^1^ Department of Clinical Pharmacology, Affiliated Hospital of Nanjing University of Chinese Medicine, Nanjing, China; ^2^ Department of Pharmacy, Affiliated Hospital of Nanjing University of Chinese Medicine, Nanjing, China; ^3^ Central Laboratory, Affiliated Hospital of Nanjing University of Chinese Medicine, Nanjing, China; ^4^ School of Medicine and Holistic Integrative Medicine, Nanjing University of Chinese Medicine, Nanjing, China

**Keywords:** Xiang-Su volatile oil, menopause, depression, network pharmacology, metabolomics

## Abstract

Menopausal depression perplexes a great number of women in later life. Xiangfu-Zisu (Xiang-Su), a traditional Chinese herbal pair composed of rhizomes of *Cyperus rotundus* L. (Xiangfu) and leaves of *Perilla frutescens* (L.) Britt. (Zisu), is frequently reported with antidepressant-like effects. The volatile oil from Xiangfu and Zisu has shown good antidepressant action, but its mechanism is still unclear. This study aimed to investigate the pharmacological mechanism of Xiang-Su (XS) volatile oil against menopausal depression through gas chromatography–mass spectrometry (GC-MS)-based network pharmacology and metabolomics. First, ADME screening was performed on actual detected components of XS volatile oil to obtain active constituents, and then duplicates of active constituent–related targets and menopausal depression–related targets were collected. These duplicates were considered as targets for XS volatile oil against menopausal depression, followed by GO and KEGG enrichment analyses. It showed that a total of 64 compounds were identified in XS volatile oil, and 38 active compounds were screened out. 42 overlapping genes between 144 compound-related genes and 780 menopausal depression–related genes were obtained. Results showed that targets of *SLC6A4* and *SLC6A3*, regulation of serotonergic and dopaminergic synapses, were involved in the antidepressant mechanism of XS volatile oil. Next, antidepressant-like effect of XS volatile oil was validated in menopausal rats by ovariectomy (OVX) combined with chronic unpredictable mild stress (CUMS). Behavioral tests, biochemical analysis, and GC-MS–based non-targeted plasma metabolomics were employed to validate the antidepressant effect of XS volatile oil. Experimental evidence demonstrated that XS volatile oil reversed behavioral parameters in the sucrose preference test (SPT), open-field test (OFT), forced swim test (FST), and serum estradiol levels in OVX rats. Furthermore, results of metabolomics indicated that XS volatile oil mainly acts on regulating metabolic pathways of phenylalanine, tyrosine and tryptophan biosynthesis, tyrosine metabolism, and tryptophan metabolism, which were corresponding with the above-predicted results. These data suggest that network pharmacology combined with metabolomics provides deep insight into the antidepressant effect of XS volatile oil, which includes regulating key targets like *SLC6A4* and *SLC6A3*, and pathways of serotonergic and dopaminergic synapses.

## Introduction

Menopause is defined as 12 months of amenorrhea following the final menstrual cycle with fluctuations of steroid hormone levels. During the menopause transition, women develop various symptoms such as sleep disturbances, hot flashes, or adverse mood, causing a high risk of depression (Tang et al., 2019, 2020; Zhou et al., 2021). However, current treatment strategies of menopausal depression including selective serotonin reuptake inhibitors (SSRIs) or hormone replacement often exhibit several adverse effects like withdrawal syndromes and sexual dysfunction in SSRIs (Khazaie et al., 2015; Moncrieff, 2019), or increased risk of cardiovascular events and breast cancer due to estrogen replacement (Anagnostis et al., 2019). Therefore, finding safe and effective drugs for the treatment of menopausal depression is of great urgency.

Traditional Chinese medicine (TCM) has a great potential to treat menopausal depression. For example, Jie-Yu Pill showed antidepressant-like effects in mice that experienced ovariectomy (OVX) with chronic unpredictable mild stress (CUMS) (Zhou et al., 2020), which was a common animal model to mimic clinical menopausal depression. Besides, essential oil from medicinal plants also has the potential to relieve depression and secondary depressive symptoms (de Sousa et al., 2017). In China, aromatic botanical drugs, rhizomes of *Cyperus rotundus* L. (Cyperaceae; *Cyperi rhizoma*, Xiangfu), leaves of *Perilla frutescens* (L.) Britt. (Lamiaceae; *Perillae folium*, Zisu), and formulas that contain these one or two botanical drugs are commonly used in treating depression or menopause-related syndrome. For example, a TCM formula Xiang-su-san (also named Koso-san in Japanese Kampo formula), which contains herbal pair Xiangfu-Zisu (Xiang-Su), had an antidepressant-like effect in mice (Ito et al., 2006). *Cyperus rotundus* L. is commonly used as a clinical herbal remedy in TCM prescription for treating depression (Zhao et al., 2015). It has also been frequently used to treat clinical gynecology disorders including premenstrual syndrome, primary dysmenorrheal, and polycystic ovary syndrome (Chen et al., 2014a, 2014b; Liao et al., 2018). Another aromatic botanical drug of *Perilla frutescens* (L.) Britt. showed antidepressant-like activities in CUMS-induced depressive mice (Yi et al., 2013; Ji et al., 2014a). Inspired by both the gynecological and neuroprotective effects of *Cyperus rotundus* L. and *Perilla frutescens* (L.) Britt., we speculate that volatile oil from the herbal pair Xiangfu-Zisu has the potential to treat menopausal depression and its following antidepressant-like effect was explored.

Network pharmacology is considered to be an appropriate approach for modern TCM pharmacological research (Zhang et al., 2019). The “compound-proteins/genes-disease” pathways are capable of describing complexities among biological systems, drugs, and diseases from a network perspective, sharing a similar holistic philosophy as TCM, and pointing to a new direction for the prediction of pharmacological mechanisms of TCM. It revealed that *Cyperus rotundus* L. showed an antidepressant effect by synergistically regulating multiple components, multiple targets, and multiple pathways through network pharmacology analysis (Jia et al., 2019). Another technology, metabolomics, makes a great contribution to understanding the basis of diseases and drug treatment. It shows advantages by integrating information from the final products of interactions among gene expression, protein function, and cellular environment (Rinschen et al., 2019). At present, metabolomics has become an important strategy for determining the antidepressant effect of TCM recipes. A classical TCM formula, Xiaoyaosan, was reported with therapeutic response in depressed patients by the metabolomics approach (Liu et al., 2015). Furthermore, the integrated strategy of metabolomics coupled with network pharmacology is an effective tool in illuminating the antidepressive action of TCM. For example, the antidepressant activity of Huang-Lian Jie-Du decoction was analyzed through network pharmacology combined with the metabolomics approach (Qu et al., 2021). However, one drawback of network pharmacology for TCM was that compounds were from some databases instead of actual identification.

**FIGURE 1:**
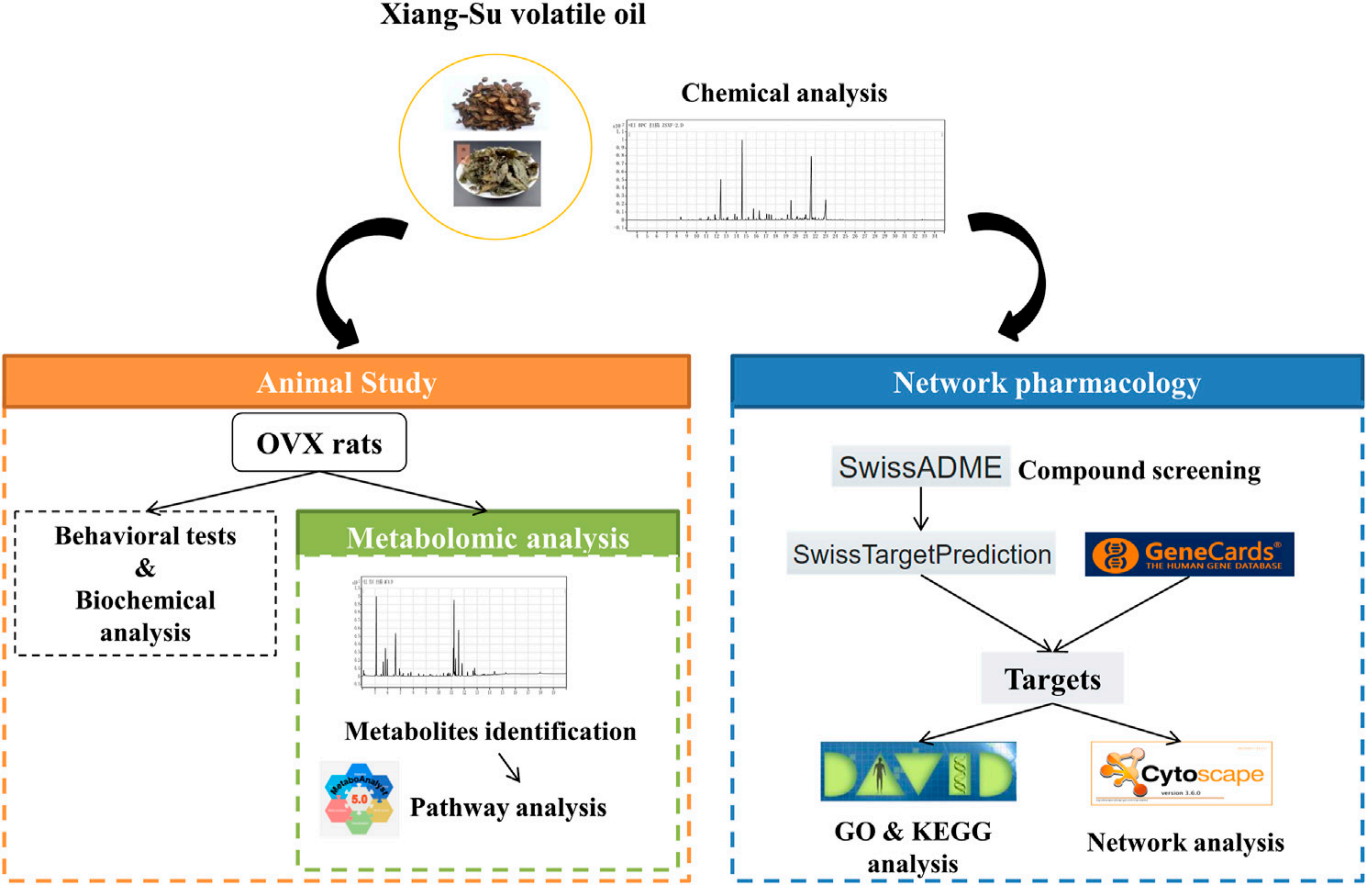
Schematic diagram of the research.

Thus, in this study, we first detected the component composition of Xiang-Su (XS) volatile oil, based on which the absorption, distribution, metabolism, and excretion (ADME) screening was carried out, and network pharmacology was utilized to predict potential bioactive compounds and elucidate the molecular mechanisms for XS volatile oil against menopausal depression. Then the antidepressant-like effect of XS volatile oil was verified in rats induced by OVX with CUMS. Finally, a gas chromatography–mass spectrometry (GC-MS)-based plasma non-targeted metabolomics approach was applied to verify the underlying antidepressant effect of XS volatile oil. The technical strategy of this study is shown in Figure 1. This study will provide experimental evidence and strengthen our understanding of the antidepressant action of XS volatile oil in menopause.

## Materials and Methods

### Materials and Reagents

The rhizomes of *Cyperus rotundus* L. (batch No. 180801) and leaves of *Perilla frutescens* (L.) Britt. (batch No. 190301) collected from Anhui were purchased from Baicaotang of Nanjing University of Chinese Medicine (Nanjing, China), subsequently authenticated by Professor Yu Zhang in Nanjing University of Chinese Medicine (Nanjing, China). Anhydrous sodium sulfate was supplied by Nanjing Chemical Reagent Co., Ltd. (Nanjing, China). Estradiol valerate tablets were purchased from Bayer HealthCare Co., Ltd. (Lot No. 580B, Guangzhou, China). Penicillin was purchased from Harbin Pharmaceutical Group Holding Co., Ltd. (Heilongjiang, China). Tween 80 and isoflurane were purchased from Shanghai Yuanye Bio-Technology Co., Ltd. (Shanghai, China). N, O-bis(trimethylsilyl)trifluoroacetamide (BSTFA), 1,2-^13^C myristic acid, pyridine, and methoxyamine hydrochloride were purchased from Sigma-Aldrich, Inc. (St. Louis, MO, United States). Methanol (chromatography grade) and *n*-hexane (chromatography grade) were supplied by Thermo Fisher Scientific Inc. (Waltham, MA, United States). The distilled water was produced by a Milli-Q purification instrument (Milford, MA, United States).

### Preparation and Compound Identification of XS Volatile Oil

#### Preparation of XS Volatile Oil

XS volatile oil consisted of two Chinese botanical drugs, namely, *Cyperus rotundus* L. and *Perilla frutescens* (L.) Britt. The volatile oil was extracted by steam distillation as follows. The rhizomes of *Cyperus rotundus* L. and leaves of *Perilla frutescens* (L.) Britt. were mixed at the ratio of 1:1 (w/w, with a total weight of 6 kg) and soaked in 8-fold of distilled water for 2 hours at room temperature, and then subjected to hydrodistillation for 3 hours to get the XS volatile oil according to the isolation procedure of volatile oil in Chinese Pharmacopoeia 2020 Edition. Later, the volatile oil was dried with anhydrous sodium sulfate and stored in brown glass at 4°C. The obtained volatile oil was weighted about 35 g. Finally, the yield of this volatile oil was 0.6% (w/w).

#### Compound Composition of XS Volatile Oil

The compound composition of XS volatile oil was detected by GC-MS using an Agilent GC 7890B-7000C system (Agilent Technologies Company, United States) fitted with an HP-5 MS capillary column (30.0 m × 250 μm × 0.25 μm, Agilent 19091S-433). GC-MS detection conditions were as follows: carrier gas, 99.999% high-purity helium; flow rate, 1.0 ml/min; sample volume, 1.0 μl (3 mg/ml XS volatile oil of *n*-hexane solution); injection port and detector temperature, 220°C; split ratio, 20:1; electronic impact, 70 eV; ion source temperature, 230°C; quadrupole rod temperature, 150°C. The oven temperature program was initially set at 50°C for 3 min, ramped at 10°C/min to 140°C, then ramped at 3°C/min to 200°C, and finally ramped at 50°C/min to 230°C and held for 2 min. Compound identification was performed by comparing the spectra in the database of the National Institute of Standards and Technology (NIST).

### Network Pharmacology

#### ADME Screening

Compounds identified from XS volatile oil were converted into the canonical simplified molecular-input line-entry system (SMILES) and screened with human gastrointestinal absorption (HIA), blood–brain barrier (BBB) permeation, and drug-likeness calculated by SwissADME (http://www.swissadme.ch/) (Daina et al., 2017). Parameters of HIA met “high,” BBB met “yes”, and two or more models among five drug-likeness models (Lipinski, Ghose, Veber, Egan, and Muegge) met “yes” were chosen as active compounds with good bioavailability.

#### Targets Linked to Identified Compounds or Menopausal Depression

Targets of the identified compounds were predicted by SwissTargetPrediction (http://www.swisstargetprediction.ch/) (Daina et al., 2019). Meanwhile, targets of menopausal depression were obtained and screened with a score ≥5.0 by retrieving the keyword of “menopausal depression” from GeneCards (https://www.genecards.org/), a database integrating all annotated and predicted genes associated with human diseases (Stelzer et al., 2016).

#### Network Construction, and GO and KEGG Enrichment Analyses

The overlapping targets between active compounds and menopausal depression were identified, and then the compound–target network was constructed and visualized by Cytoscape 3.6.0. Degree, betweenness centrality, and closeness centrality indicating the topological importance of nodes in the network were analyzed by Network Analyzer Tool in Cytoscape (Otasek et al., 2019). Gene Ontology (GO) and Kyoto Encyclopedia of Genes and Genomes (KEGG) pathway enrichment analyses were performed using the online functional annotation and enrichment tool DAVID (https://david.ncifcrf.gov/) (Huang et al., 2009), and plotted by the online platform for data analysis and visualization (http://www.bioinformatics.com.cn/). GO terms and KEGG pathways with a *p*-value < 0.05 were considered statistically significant.

### Animal Study

#### Animals and Treatment

**FIGURE 2:**
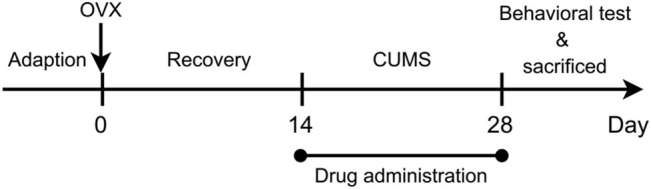
Schematic representation of the experimental procedure.

Female SD rats (180–200 g, supplied by Nantong University, Jiangsu, China) were housed and fed with unlimited access to food and water under a 12-hour light/dark cycle except for the following CUMS procedure. After adaptive feed, the rats were randomly divided into six groups as follows: sham group (negative control), OVX group (model control), OVX with estradiol valerate group (positive control, OVX-E2, 0.18 mg/kg), and OVX with different doses of XS volatile oil (OVX-XSL, 10.8 mg/kg; OVX-XSM, 32.4 mg/kg; OVX-XSH, 97.2 mg/kg) groups. The dose of XS volatile oil was converted by a drug-extract ratio of 0.6% (w/w) from the recommended dose of *Cyperus rotundus* L. and *Perilla frutescens* (L.) Britt for human in Chinese Pharmacopoeia 2020 Edition, and set in a safe dose range according to references (Jebasingh et al., 2012; Ji et al., 2014a; Kum et al., 2017; Kangwan et al., 2019). XS volatile oil was dissolved in distilled water with 0.5% Tween 80 to form an emulsion with a concentration of 2.0, 6.0, and 18.0 mg/ml, respectively, with gastric perfusion in a volume of 5.4 ml/kg once daily. Meanwhile, distilled water with 0.5% Tween 80 was used for the negative and model control groups. Estradiol valerate tablets used for the positive control group were dissolved in distilled water to a concentration of 0.036 mg/ml of estradiol valerate with gastric perfusion in a volume of 5.0 ml/kg. Bilateral OVX was performed in the rats in model control, positive control, and three XS volatile oil groups, respectively, while pseudo-operation was performed in the sham group. All surgical procedures were performed under isoflurane inhalation anesthesia. After surgery, penicillin was injected for three consecutive days, and all rats were kept in separate cages. Then, all OVX rats were experienced with the CUMS procedure, which was followed by Willner et al. with minor modifications (Willner et al., 1987). Random stressors included food or water deprivation (12 h), cage tilt (12 h), wet bedding (12 h), empty cage (12 h), tail pinch (2 min), physical restraint (2 h), light (24 h), stroboscopic light (12 h), and intermittent light (12 h). The animal study was reviewed and approved by the Ethical Committee of Jiangsu Province Hospital of Chinese Medicine and strictly followed the guidelines for the care and use of laboratory animals (National Research Council Committee for the Update of the Guide for the and Use of Laboratory, 2011). All the experimental procedures are shown in Figure 2.

#### Behavioral Tests

Sucrose preference test (SPT): Before the test, rats were trained for 3 days to consume from two bottles of sucrose solution (1%, w/v) or one bottle of distilled water and one bottle of sucrose. The test was conducted after the rats were water-deprived for 18 hours. Two bottles of water and sucrose were given to rats for 1 hour, then following 1 hour with interchanged placement. Finally, the consumed weight was recorded, and the following formula was used to calculate the sucrose preference rate: sucrose preference rate (%) = sucrose consumption/(water consumption + sucrose consumption) × 100. The SPT was performed according to our previous study with minor modifications (Jing et al., 2019).

Forced swim test (FST): The day before the test, rats were forced to pre-swim for 15 min individually in plastic cylinders filled with water at a temperature of about 25°C to a depth of 30 cm. The cylinder was cleaned carefully after each test. After 24 hours, the immobile time of rats during 5 min of FST was recorded. Immobile time refers to the time during which rats stopped struggling, floated motionlessly, or only tried to keep their heads above water. The test session was recorded by a video camera and analyzed using the SuperFst system (XinRuan Inc., Ltd., Shanghai, China). The FST was performed according to the previous study with minor modifications (Pan et al., 2021).

Open-field test (OFT): The OFT was performed in an open-field apparatus (100 cm × 100 cm). All rats were placed individually in the center of the apparatus. The TopScanHR system (CleverSys Inc, Reston, VA, United States) was used to record the moving distance during 5 min in the apparatus. At each test interval, ethyl alcohol was used to clean the apparatus to avoid excretion interference. The OFT was performed according to the previous study with minor modifications (Pan et al., 2021).

#### Biochemical Analysis

Before all rats were sacrificed, blood was first collected from the orbital sinus, and the serum was obtained by centrifugation (3,000 r/min, 15 min). Then the supernatant was separated and stored at −80°C before use. The serum levels of estradiol were measured using an enzyme-linked immunosorbent assay kit (Lot No. ZC-36464, ZCIBIO Technology Co. Ltd., Shanghai, China) according to the manufacturer’s instructions. Briefly, the serum samples were first diluted five times for measurement. Then 50 μl of serum samples as well as standards were added in a 96-well plate precoated with target antibody and followed by adding 100 μl horseradish peroxidase–conjugated antibody. After incubation for 60 min at 37°C, the liquid was discarded. Subsequently, each well was washed with 350 μl washing buffer for five times. 50 μl of substrate A and substrate B were then added into each well, which was next incubated at 37°C in dark for 15 min. Finally, 50 μl of termination solution was added to each well, and the absorbance was measured at 450 nm by a microplate reader. The serum estradiol levels were calculated according to the standard curve.

#### Sample Preparation for GC-MS Metabolomics

Blood was also collected in heparin tubes from the abdominal aorta after isoflurane inhalation anesthesia and centrifuged to separate plasma for metabolomic analysis. The optimized method was based on the derivatization method by BSTFA. 50 μl of plasma was accurately taken and added with 200 μl methanol with 12.5 μg/ml 1,2-^13^C myristic acid as an internal standard. After 3-min vortex and centrifugation at 4°C, 18,000 r/min for 10 min, 150 μl supernatant was separated and concentrated in a low-temperature centrifugal concentrator (50°C, 2 hours) until the solvent was completely evaporated. The residue was dissolved in 45 μl of 10 mg/ml methoxyamine hydrochloride in a pyridine solution. After 5-min vortex and oscillation at 30°C for 1.5 hours (450 r/min), 45 μl of derivatization reagent BSTFA was precisely added. Then the sample was oscillated at 37°C for 0.5 hours (450 r/min) to full derivatization. Finally, the supernatant was collected after centrifugation at 4°C, 18,000 r/min for 10 min. The quality control (QC) sample was prepared by mixing the remaining supernatant after methanol precipitation of all samples, and the following operations were the same as described before. The QCs were injected at regular intervals (every 10 samples) to monitor the stability of the analytical process.

#### GC-MS Analysis

The derivatized plasma samples were analyzed on the same equipment as compound identification of XS volatile oil. The detection conditions were as follows: carrier gas; 99.999% high-purity helium; flow rate, 1.0 ml/min; injection volume, 1.0 μl; injector temperature, 240°C; electronic impact, 70 eV; ion source temperature, 300°C; quadrupole rod temperature, 180°C. The column temperature was initially set at 60°C and held for 1 min, increased to 240°C at a rate of 20°C/min, then increased to 260°C at a rate of 5°C/min, and finally increased to 320°C at a rate of 30°C/min and held for 4 min. The solvent delay time was 3.95 min.

### Data Analysis

The GC-MS original data were processed through Agilent MassHunter Workstation software (Qualitative Analysis B.07.00). The database of NIST was used to characterize the metabolites. Then the data were normalized, and names, retention time, peak intensity, and mass of all samples were processed with the Microsoft Excel software (version 2007). Principal component analysis (PCA) and orthogonal partial least square discriminant analysis (OPLS-DA) were performed on the data by SIMCA-P 14.1 software. We also used the online tool Metaboanalyst 5.0 (https://www.metaboanalyst.ca/home.xhtml) to assist in finding metabolite differences and constructing metabolic pathways. All values were presented as the mean ± standard error of the mean (SEM). In the behavioral tests and biochemical analysis, the data were analyzed by one-way ANOVA followed by Fisher’s LSD test through GraphPad 6.0 software. *p*-value < 0.05 was considered statistically significant.

## Results

### Compound Identification of XS Volatile Oil and Active Component Screening

The actual chemical composition of XS volatile oil is shown in Table 1, and its chromatographic profile is shown in Supplementary Figure S1. The proportion of each component was obtained by peak area normalization. A total of 64 compounds were identified, accounting for 99.67% area of all the detected chromatographic peaks in XS volatile oil. Among them, perilla aldehyde (C11, 12.08%) from *Perilla frutescens* (L.) Britt., cyperenone (C54, 27.14%), cyperene (C19, 16.05%), α-cyperone (C60, 9.27%), and dehydrofukinone (C45, 4.95%) from *Cyperus rotundus* L. were the top 5 major components in this volatile oil, accounting for nearly 70% area of all the peaks. Then, we screened out 38 active compounds according to the parameters of HIA, BBB permeation, and drug-likeness in ADME screening (Table 2). Among these active compounds, perilla aldehyde (C11), dehydrofukinone (C45), cyperenone (C54), and α-cyperone (C60) were also included, which might account for the bioactivity of XS volatile oil.

**TABLE 1:** Chemical composition of XS volatile oil.

No	t_R_/min	Compound	Formula	%	Reverse match
C1	8.41	d-Limonene	C_10_H_16_	1.15	892
C2	9.60	Linalool	C_10_H_18_O	0.14	878
C3	10.30	L-Pinocarveol	C_10_H_16_O	0.36	908
C4	11.00	Verbenone	C_10_H_14_O	0.09	801
C5	11.09	α-Terpineol	C_10_H_18_O	0.22	904
C6	11.19	(-)-Myrtenol	C_10_H_16_O	0.76	867
C7	11.40	Berbenone	C_10_H_14_O	0.17	907
C8	11.64	β-Cyclocitral	C_10_H_16_O	0.07	846
C9	11.85	p-Cumic aldehyde	C_10_H_12_O	1.01	908
C10	11.95	1-(Furan-2-yl)-4-methylpentan-1-one	C_10_H_14_O_2_	0.47	913
C11	12.44	Perilla aldehyde	C_10_H_14_O	12.08	920
C12	12.54	8-(1-Methylethylidene)bicycle[5.1.0]octane	C_11_H_18_	0.24	866
C13	12.72	Perilla alcohol	C_10_H_16_O	0.35	911
C14	13.15	Cyprotene	C_14_H_24_	0.47	883
C15	13.62	Epoxycaryophyllene	C_15_H_24_O	0.13	809
C16	13.86	2,4-Patchouladiene	C_15_H_22_	1.01	931
C17	13.95	(+)-Cyclosativene	C_15_H_24_	0.08	893
C18	14.08	Nootkatene	C_15_H_22_	0.66	858
C19	14.60	Cyperene	C_15_H_24_	16.05	951
C20	14.93	β-Caryophyllene	C_15_H_24_	0.20	917
C21	14.97	9,10-dehydro-Isolongifolene	C_15_H_22_	0.12	725
C22	15.23	Cypera-2,4(15)-diene	C_15_H_22_	0.56	928
C23	15.50	α-Selinene	C_15_H_24_	0.26	848
C24	15.75	Rotundene	C_15_H_24_	2.30	910
C25	16.00	β-Vetispirene	C_15_H_22_	0.12	850
C26	16.04	β-Gurjunene	C_15_H_24_	0.27	890
C27	16.32	β-Selinene	C_15_H_24_	2.23	935
C28	16.43	(+)-Valencene	C_15_H_24_	0.29	943
C29	16.55	15-Hydroxy-α-muurolene	C_15_H_24_O	0.12	805
C30	16.62	(+)-Isovalencenol	C_15_H_24_O	0.12	857
C31	16.90	(-)-Nootkatene	C_15_H_22_	0.28	887
C32	17.07	γ-Gurjunene	C_15_H_24_	1.48	822
C33	17.30	Epoxycyperene	C_15_H_24_O	1.29	888
C34	17.54	α-Calacorene	C_15_H_20_	1.20	925
C35	17.80	10-Epi-Acora-3, 11-dien-15-al	C_15_H_22_O	0.12	821
C36	17.99	Spathulenol	C_15_H_24_O	0.41	780
C37	18.24	Aristol-1(10)-en-9-ol	C_15_H_24_O	0.27	823
C38	18.52	1, 3-Di(propen-1-yl)adamantane	C_16_H_24_	0.28	775
C39	18.56	Caryophyllene oxide	C_15_H_24_O	0.10	902
C40	18.64	(-)-Spathulenol	C_15_H_24_O	0.41	800
C41	18.81	Eudesma-4(15), 7-dien-1β -ol	C_15_H_24_O	0.18	802
C42	18.96	4,6-diisopropylidene-8,8-dimethyl-Bicyclo[5.1.0]octan-2-one	C_16_H_24_O	0.13	752
C43	19.18	*cis*-α-Copaene-8-ol	C_15_H_24_O	1.65	837
C44	19.34	4,4,11,11-tetramethyl-7-Tetracyclo[6.2.1.0(3.8)0(3.9)]undecanol	C_15_H_24_O	0.10	824
C45	19.53	Dehydrofukinone	C_15_H_22_O	4.95	837
C46	19.65	γ-Gurjunenepoxide-(2)	C_15_H_24_O	0.28	823
C47	19.89	Caryophylladienol II	C_15_H_24_O	0.13	845
C48	20.10	13-nor-Eremophil-1(10)-en-11-one	C_14_H_22_O	0.58	835
C49	20.16	3a,7,7-Trimethyltetrahydro-1H-cyclopropa[c]indene-2,3(1ah,3ah)-dione	C_13_H_18_O_2_	0.73	838
C50	20.44	Isoaromadendrene epoxide	C_15_H_24_O	0.51	835
C51	20.90	Calarene epoxide	C_15_H_24_O	0.66	818
C52	21.02	Mustakone	C_15_H_22_O	1.75	893
C53	21.26	Hexahydro-2,5,5-trimethyl-2H-,4a-ethanonaphthalen-8(5H)-one	C_15_H_24_O	0.38	821
C54	21.58	Cyperenone	C_15_H_22_O	27.14	923
C55	21.72	α-Costal	C_15_H_22_O	0.51	875
C56	21.80	(-)-Rotundone	C_15_H_22_O	0.21	906
C57	21.98	6-Isopropenyl-4,8a-dimethyl-3,5,6,7,8,8a-hexahydro-2(1H)-naphthalenone	C_15_H_22_O	0.67	834
C58	22.33	Cyclocopacamphan-12-ol	C_15_H_24_O	0.60	789
C59	22.82	Zizanal	C_15_H_22_O	0.50	835
C60	23.04	α-Cyperone	C_15_H_22_O	9.27	923
C61	23.22	Isovelleral	C_15_H_20_O_2_	0.21	875
C62	23.40	Aristolone	C_15_H_22_O	0.14	867
C63	24.50	Nootkatone	C_15_H_22_O	0.27	913
C64	24.74	Furopelargone A	C_15_H_22_O_2_	0.18	811

**TABLE 2:** Compounds of XS volatile oil after ADME screening.

No	Compound	Formula	SMILES
C2	Linalool	C_10_H_18_O	CC(=CCCC(C)(C=C)O)C
C3	L-Pinocarveol	C_10_H_16_O	CC1(C2CC1C(=C)C(C2)O)C
C4	Verbenone	C_10_H_14_O	CC1=CC(=O)C2CC1C2(C)C
C5	α-Terpineol	C_10_H_18_O	CC1=CCC(CC1)C(C)(C)O
C6	(-)-Myrtenol	C_10_H_16_O	CC1(C2CC=C(C1C2)CO)C
C7	Berbenone	C_10_H_14_O	CC1=CC(=O)[C@@H]2CC1C2(C)C
C8	β-Cyclocitral	C_10_H_16_O	CC1=C(C(CCC1)(C)C)C=O
C9	p-Cumic aldehyde	C_10_H_12_O	CC(C)C1=CC=C(C=C1)C=O
C10	1-(Furan-2-yl)-4-methylpentan-1-one	C_10_H_14_O_2_	CC(C)CCC(=O)C1=CC=CO1
C11	Perilla aldehyde	C_10_H_14_O	CC(=C)C1CCC(=CC1)C=O
C13	Perilla alcohol	C_10_H_16_O	CC(=C)C1CCC(=CC1)CO
C15	Epoxycaryophyllene	C_15_H_24_O	CC1(CC2C1CCC3(C(O3)CCC2=C)C)C
C30	(+)-Isovalencenol	C_15_H_24_O	CC1CCC=C2C1(CC(=C(C)CO)CC2)C
C33	Epoxycyperene	C_15_H_24_O	CC1CCC2CC34C1(C2(C)C)CCC3(O4)C
C36	Spathulenol	C_15_H_24_O	CC1(C2C1C3C(CCC3(C)O)C(=C)CC2)C
C37	Aristol-1(10)-en-9-ol	C_15_H_24_O	CC1CC(CC2=C(CCC12)C)C=C(C)CO
C39	Caryophyllene oxide	C_15_H_24_O	CC1(CC2C1CCC3(C(O3)CCC2=C)C)C
C40	(-)-Spathulenol	C_15_H_24_O	CC1(C2C1C3C(CCC3(C)O)C(=C)CC2)C
C43	*cis*-α-Copaene-8-ol	C_15_H_24_O	CC1=CCC2C3C1C2(CC(C3C(C)C)O)C
C45	Dehydrofukinone	C_15_H_22_O	CC1CCCC2=CC(=O)C(=C(C)C)CC12C
C46	γ-Gurjunenepoxide-(2)	C_15_H_24_O	CC1CCC(C=C2C1CCC2C)C3(CO3)C
C47	Caryophylladienol II	C_15_H_24_O	CC1(CC2C1CCC(=C)C(CCC2=C)O)C
C48	13-nor-Eremophil-1(10)-en-11-one	C_14_H_22_O	CC1CCC=C2C1(CC(CC2)C(=O)C)C
C49	3a,7,7-Trimethyltetrahydro-1H-cyclopropa[c]indene-2,3(1ah,3ah)-dione	C_13_H_18_O_2_	CC1(CCCC2(C13CC3C(=O)C2=O)C)C
C50	Isoaromadendrene epoxide	C_15_H_24_O	CC1CCC2C1C3C(C3(C)C)CC4C2(O4)C
C51	Calarene epoxide	C_15_H_24_O	CC1CCC2C3(C1(C4C(C4(C)C)CC3)C)O2
C52	Mustakone	C_15_H_22_O	CC1=CC(=O)C2C3C1C2(CCC3C(C)C)C
C54	Cyperenone	C_15_H_22_O	CC1CCC2CC3=C(C(=O)CC13C2(C)C)C
C55	α-Costal	C_15_H_22_O	CC1=CCCC2(C1CC(CC2)C(=C)C=O)C
C56	(-)-Rotundone	C_15_H_22_O	CC1CCC(CC2=C1C(=O)CC2C)C(=C)C
C57	6-Isopropenyl-4,8a-dimethyl-3,5,6,7,8,8a-hexahydro-2(1H)-naphthalenone	C_15_H_22_O	CC1=C2CC(CCC2(CC(=O)C1)C)C(=C)C
C58	Cyclocopacamphan-12-ol	C_15_H_24_O	CC(CO)C1CCC2(C3C1C4C2(C4C3)C)C
C59	Zizanal	C_15_H_22_O	CC1(C2CCC3(C2)C(CCC3C1=C)C=O)C
C60	α-Cyperone	C_15_H_22_O	CC1=C2CC(CCC2(CCC1=O)C)C(=C)C
C61	Isovelleral	C_15_H_20_O_2_	CC1(CC2C=C(C3(CC3(C2C1)C)C=O)C=O)C
C62	Aristolone	C_15_H_22_O	CC1CCCC2=CC(=O)C3C(C12C)C3(C)C
C63	Nootkatone	C_15_H_22_O	CC1CC(=O)C=C2C1(CC(CC2)C(=C)C)C
C64	Furopelargone A	C_15_H_22_O_2_	CC1CCC(C1C2=C(C=CO2)C(C)C)C(=O)C

### Targets and Network Analysis of XS Volatile Oil Against Menopausal Depression

**FIGURE 3:**
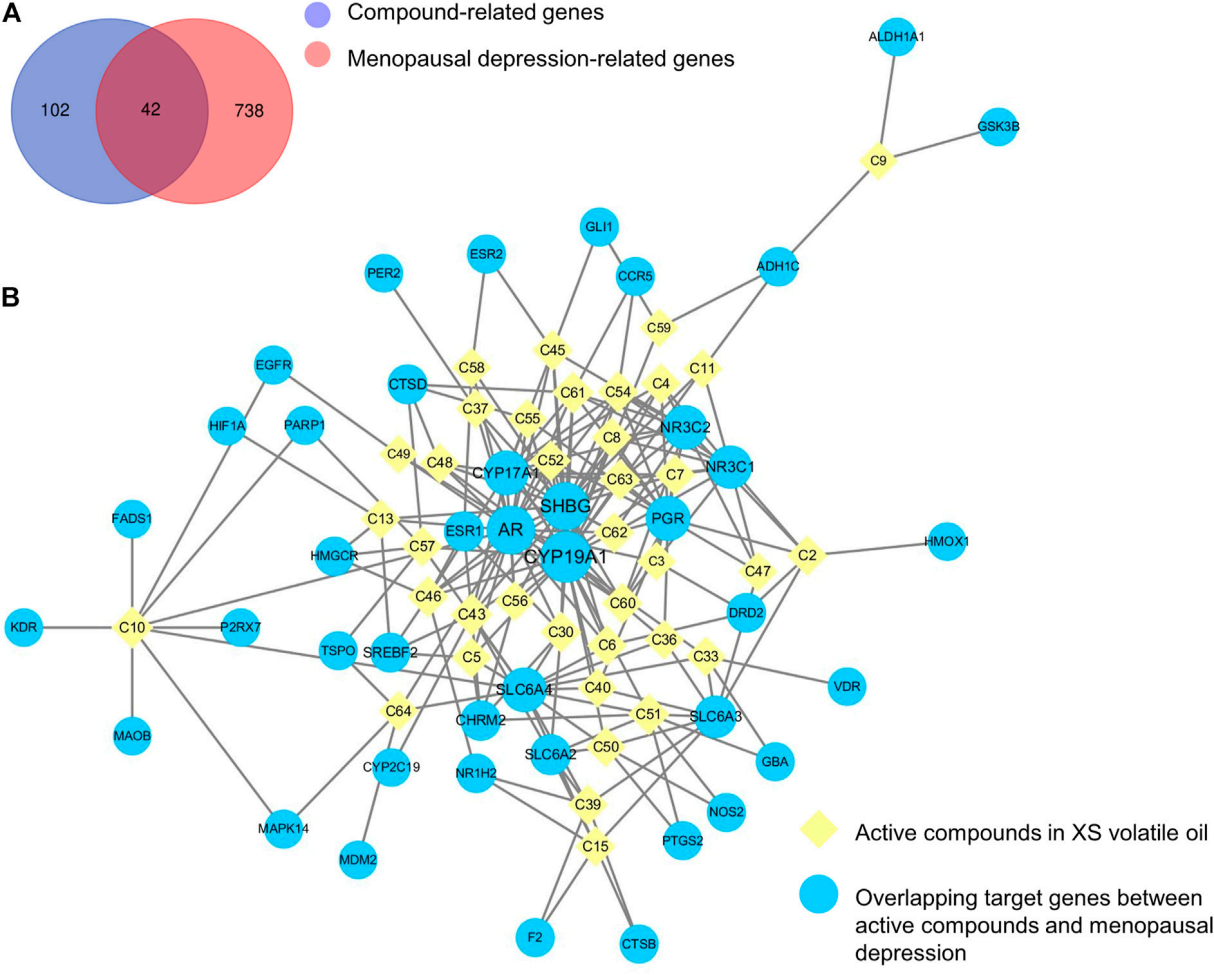
Venn diagram of overlapping genes between compound and menopausal depression–related genes **(A)** and compound–target network of XS volatile oil against menopausal depression **(B)**.

The SMILES of 38 abovementioned active compounds were plotted into SwissTargetPrediction to obtain the potential targets of each compound. Then 144 compound-related genes were collected after removing the duplicates (Supplementary Table S1). Besides, a total of 780 genes related to menopausal depression (score ≥ 5.0) were retrieved from the GeneCards database (Supplementary Table S2). Results showed that there were 42 overlapping genes by matching 144 compound-related genes with 780 disease-related genes (Figure 3A; Supplementary Table S3). A compound–target network of XS volatile oil against menopausal depression was constructed and visualized by importing 38 active compounds and 42 overlapping genes into Cytoscape 3.6.0. This network contained 80 nodes and 216 edges (Figure 3B). The contribution difference of these active compounds and genes to XS volatile oil against menopausal depression was shown in this network. According to the parameters of degree, betweenness centrality, and closeness centrality by topological analysis, *cis*-α-copaene-8-ol (C43), 1-(furan-2-yl)-4-methylpentan-1-one (C10), isovelleral (C61), calarene epoxide (C51), γ-gurjunenepoxide-(2) (C46), cyperenone (C54), dehydrofukinone (C45), (+)-isovalencenol (C30), α-cyperone (C60), and aristolone (C62) were the top 10 ingredient nodes linked to more targets. In addition, several targets such as cytochrome P450 family 19 subfamily A member 1 (*CYP19A1*), cytochrome P450 family 17 subfamily A member 1 (*CYP17A1*), solute carrier family six-member 4 (*SLC6A4*), and solute carrier family six-member 3 (*SLC6A3*) were considered as the crucial targets of XS volatile oil against menopausal depression ranked by degree, betweenness centrality, and closeness centrality.

### GO and KEGG Enrichment Analyses of XS Volatile Oil Against Menopausal Depression

**FIGURE 4:**
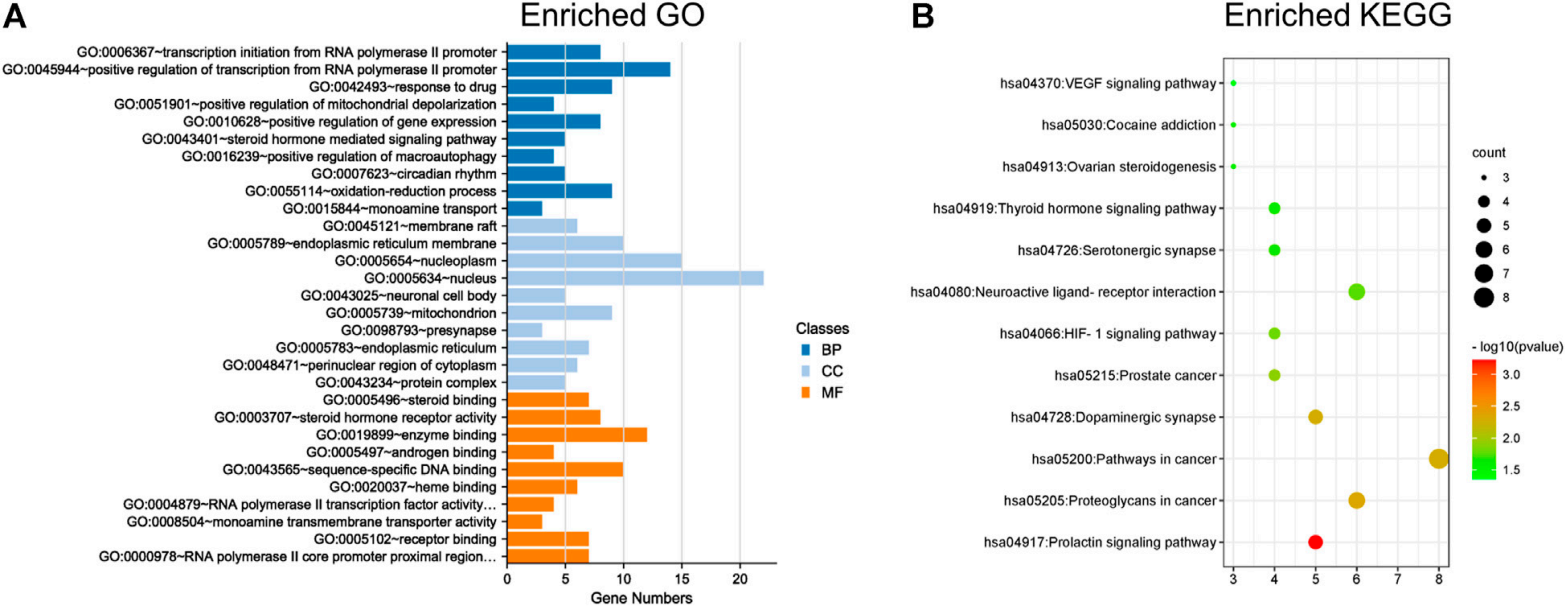
GO and KEGG enrichment analyses of 42 targets for XS volatile oil against menopausal depression. **(A)** Histogram of the top 10 terms in GO enrichment analysis. **(B)** Bubble chart of 12 signaling pathways in KEGG enrichment analysis. BP, biological process; CC, cellular component; MF, molecular function.

GO and KEGG enrichment analyses were performed on the abovementioned 42 targets for the treatment of XS volatile oil against menopausal depression. As shown in Figure 4A, the top 10 terms in the GO biological process (BP), cellular component (CC), and molecular function (MF) were ranked by *p*-value (Supplementary Table S4). It showed that these potential targets were mainly located in the neuronal cell body and pre-synapse, regulating molecular functions like steroid binding, enzyme binding, and monoamine transmembrane transporter activity, and participated in biological processes like steroid hormone-mediated signaling pathway and monoamine transport. Furthermore, the results of KEGG analysis indicated that 42 overlapping genes of XS volatile oil against menopausal depression were significantly enriched in 12 signaling pathways (*p* < 0.05), of which dopaminergic synapse, serotonergic synapse, and ovarian steroidogenesis were involved (Figure 4B; Supplementary Table S5). The crucial genes like *CYP19A1* and *CYP17A1* were involved in ovarian steroidogenesis signaling pathways, *SLC6A4* was related to the serotonergic synapse, and *SLC6A3* was related to the dopaminergic synapse. These data suggested that XS volatile oil may exert its antidepressant effect by regulating monoamine transport and pathways of dopaminergic and serotonergic synapses.

### XS Volatile Oil Prevented Depressive-like Behaviors in OVX Rats

**FIGURE 5:**
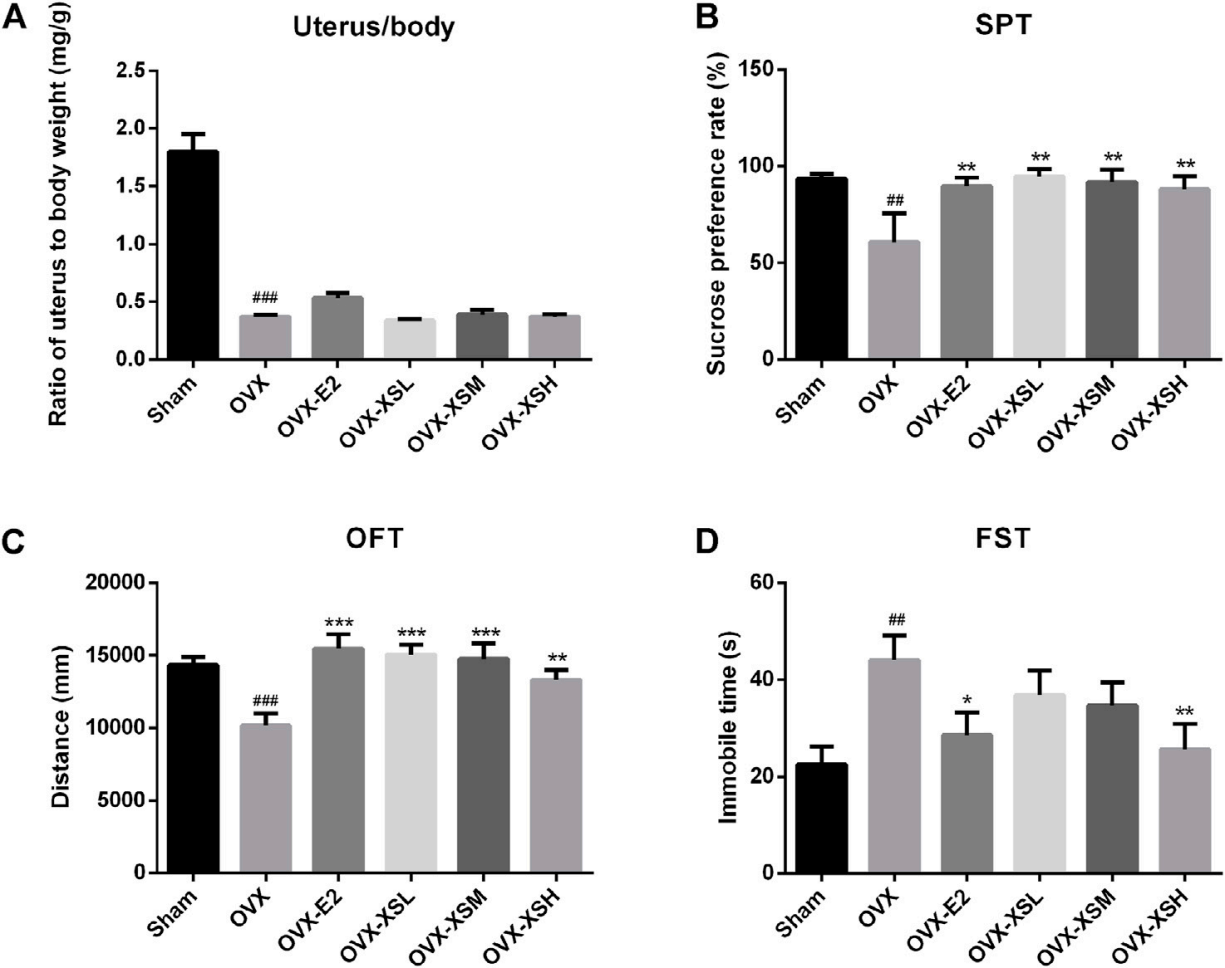
Effects of XS volatile oil and estradiol treatment on behavior parameters in OVX rats. **(A)** Ratio of uterus to body weight. **(B)** Sucrose preference test (SPT). **(C)** Open-field test (OFT). **(D)** Forced swim test (FST). Data were represented as mean ± SEM; *n* = 6–10 per group. OVX-E2 (estradiol valerate, 0.18 mg/kg), OVX-XSL (10.8 mg/kg), OVX-XSM (32.4 mg/kg), OVX-XSH (97.2 mg/kg). ^##^ *p* < 0.01, ^###^ *p* < 0.001 compared with the sham group; ^*^ *p* < 0.05, ^**^ *p* < 0.01, ^***^ *p* < 0.001 compared with the OVX group.

As shown in Figure 5A, OVX resulted in a significant decrease in the ratio of the uterus to body weight (*p* < 0.001) compared to sham surgery. Behavioral tests of SPT, OFT, and FST were performed to determine the antidepressant effect of XS volatile oil. OVX rats showed depressive-like behaviors, including significantly decreased sucrose consumption in SPT (*p* < 0.01) (Figure 5B), reduced total traveled distance in OFT (*p* < 0.001) (Figure 5C), and increased immobile time in FST (*p* < 0.01) (Figure 5D), compared with the sham group. OVX rats with the administration of XS volatile oil (10.8, 32.4, 97.2 mg/kg) expressed significantly increased sucrose preference (*p* < 0.01) and traveled distance (10.8 and 32.4 mg/kg, *p* < 0.001; 97.2 mg/kg, *p* < 0.01). Additionally, immobile time in the high-dose group of XS volatile oil (97.2 mg/kg) was reduced compared with the OVX rats. These data showed the antidepressant effect of XS volatile oil in OVX rats. The positive drug estradiol (0.18 mg/kg) also presented antidepressant effect by improving sucrose preference in SPT (*p* < 0.01), increasing total traveled distance in OFT (*p* < 0.001), and decreasing immobile time in FST (*p* < 0.05).

### XS Volatile Oil Alleviated Serum Estradiol Deficiency in OVX Rats

**FIGURE 6:**
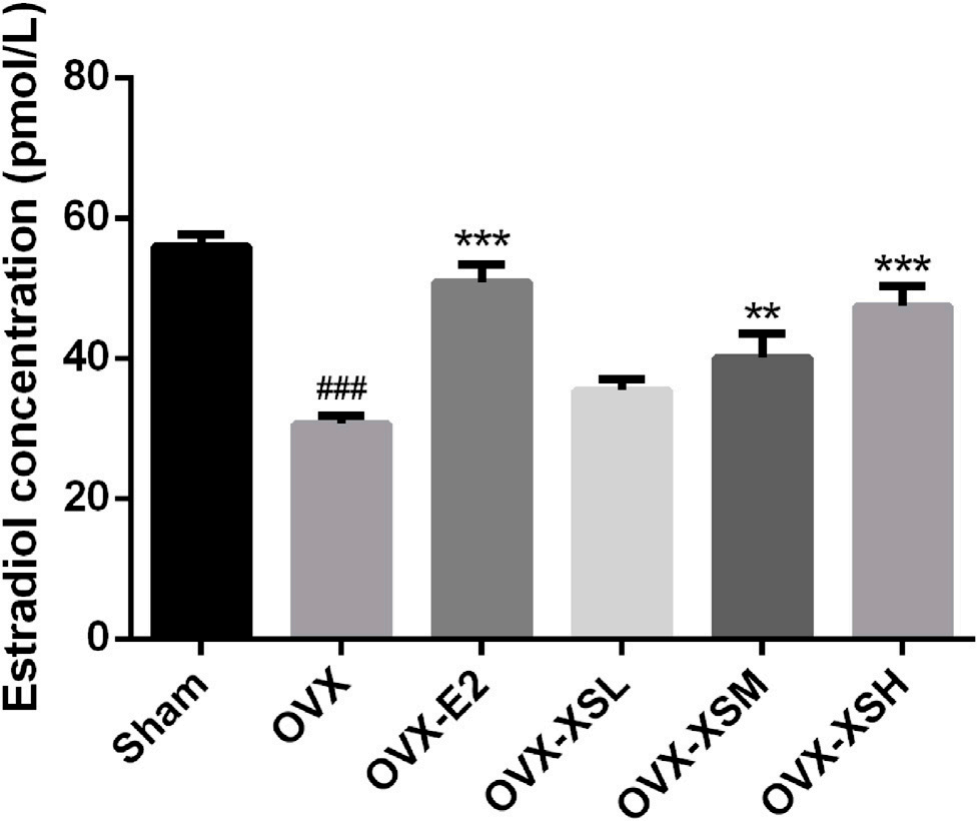
Effects of XS volatile oil and estradiol treatment on serum estradiol levels in OVX rats. Data were represented as mean ± SEM; *n* = 8–10 per group. OVX-E2 (estradiol valerate, 0.18 mg/kg), OVX-XSL (10.8 mg/kg), OVX-XSM (32.4 mg/kg), OVX-XSH (97.2 mg/kg). ^###^ *p* < 0.001 compared with the sham group; ^**^ *p* < 0.01, ^***^ *p* < 0.001 compared with the OVX group.

The levels of serum estradiol decreased markedly in OVX rats compared with the sham rats (*p* < 0.001). Administration of XS volatile oil remarkably increased the serum estradiol levels in a dose-dependent manner (32.4 mg/kg, *p* < 0.01; 97.2 mg/kg, *p* < 0.001), while estradiol had the same regulatory effect (*p* < 0.001) in OVX rats (Figure 6).

### XS Volatile Oil Improved Metabolic Profiles in OVX Rats

**FIGURE 7:**
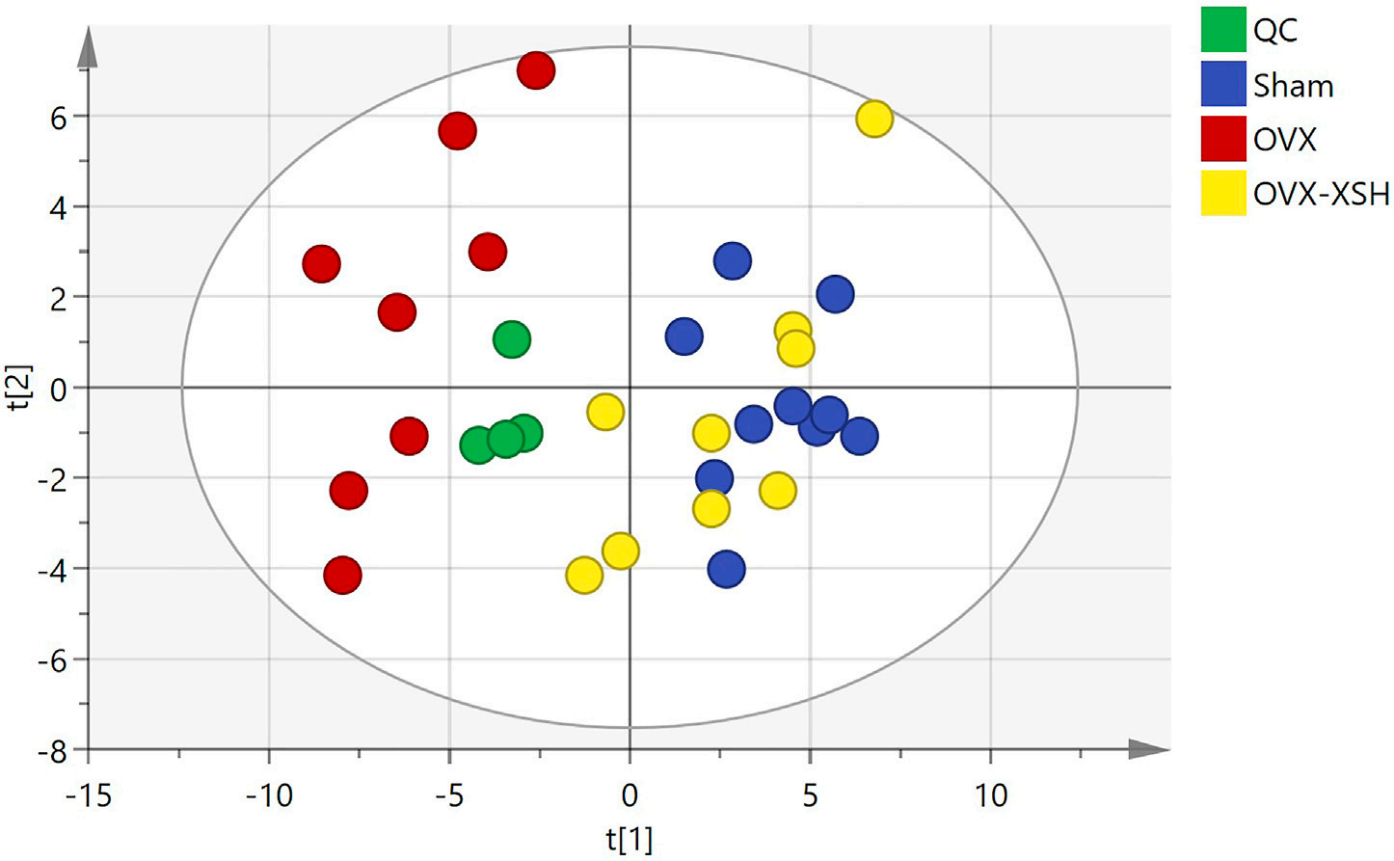
Score plot of PCA. *n* = 8–10 per group, QC (quality control), and OVX-XSH (97.2 mg/kg).

**FIGURE 8:**
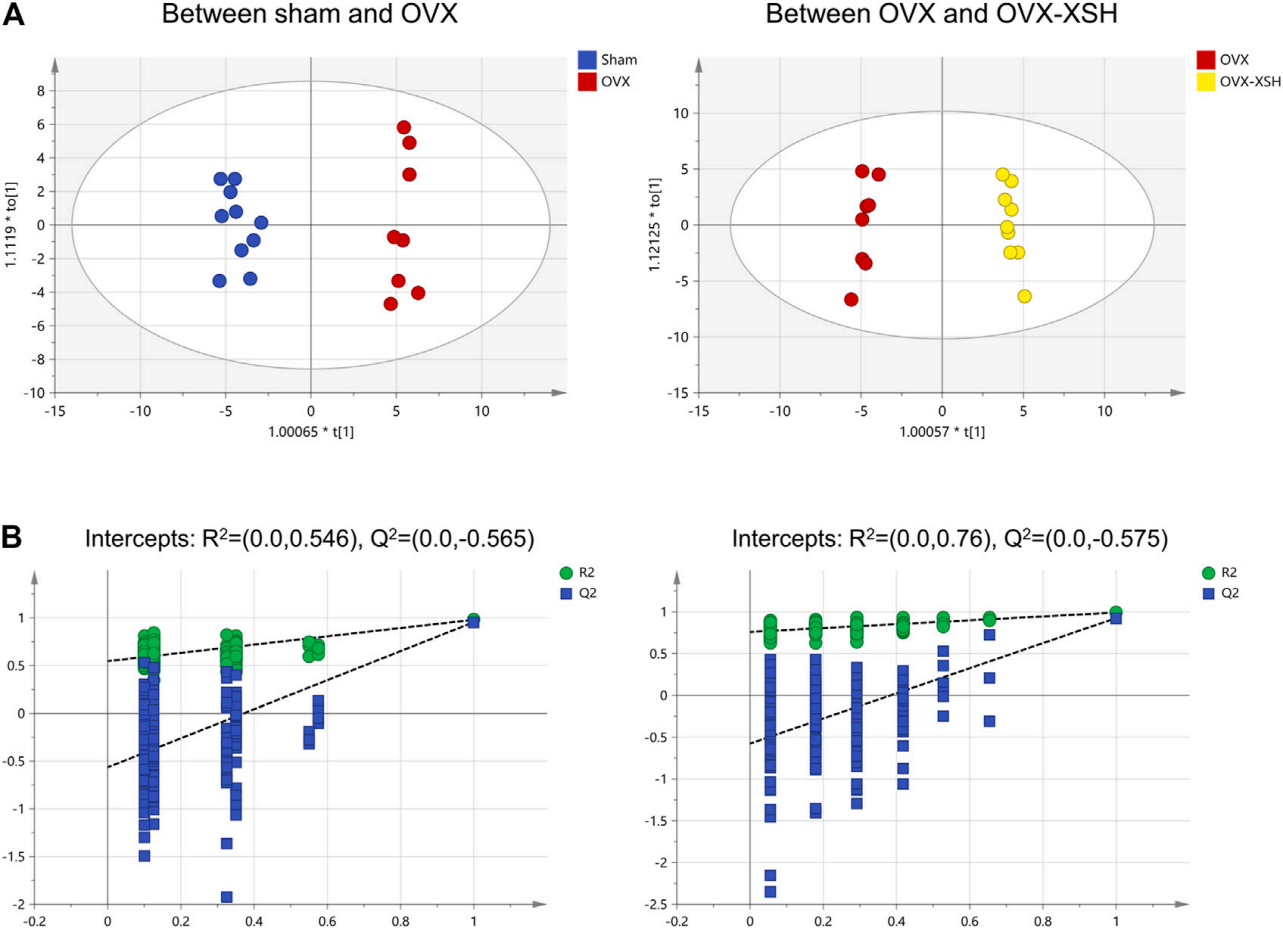
Multivariate statistical analysis of metabolic characters of plasma samples acquired by GC-MS. **(A)** Score plot of OPLS-DA between different animal groups. **(B)** Results of cross-validation between different animal groups. *n* = 8–10 per group.

The unsupervised PCA was used to check the quality of data for the metabolomic analysis. As shown in Figure 7, samples from the sham, OVX, OVX-XSH, and QC groups were within the 95% Hotelling’s T-squared ellipse and were separated into clusters (R^2^X = 0.723, Q^2^ = 0.375). No outlier was found among these samples. The supervised OPLS-DA was performed to identify the metabolites responsible for the separation between OVX and the other two groups. All groups in the OPLS-DA models have met the 95% Hotelling’s T-squared ellipse and showed clear separation (Figure 8A). The 200 times permutation test was conducted to assess the predictive accuracy and statistical significance. Results from cross-validation suggested the model showed good predictability (Figure 8B). The metabolite peaks with VIP > 1.0 were selected.

**FIGURE 9:**
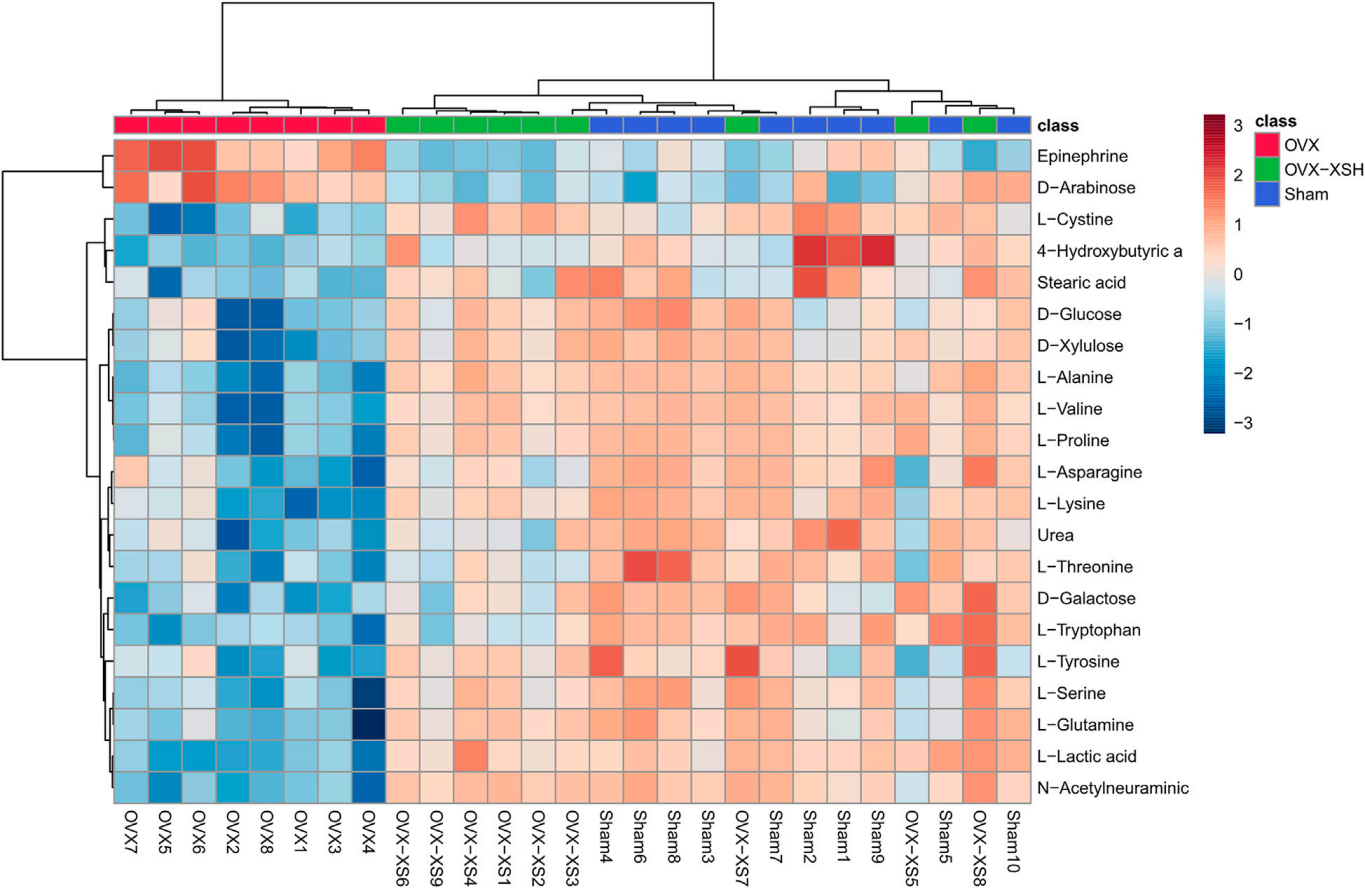
Discriminating metabolites differentiated in sham, OVX, and OVX-XSH groups. FC > 1.2 or <0.83, *p*-value < 0.05 and VIP-value > 1.0, *n* = 8–10 per group, OVX-XSH (97.2 mg/kg).

**FIGURE 10:**
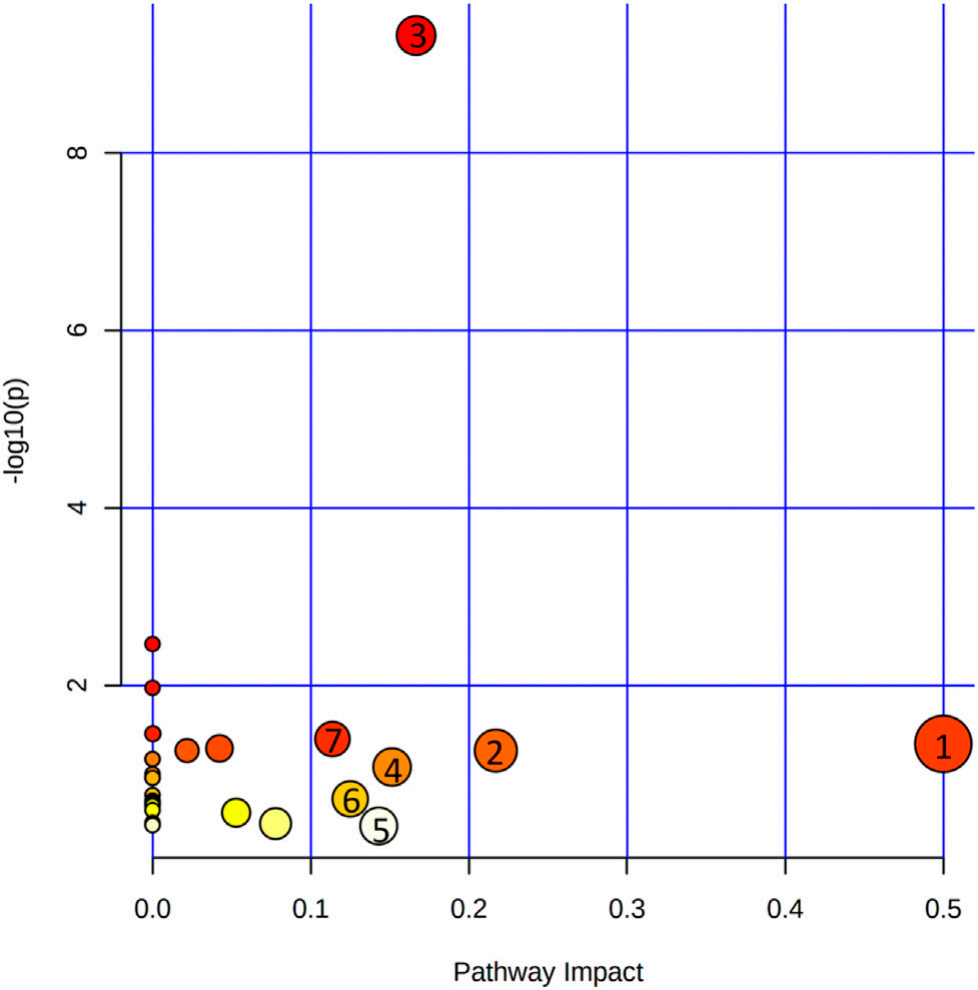
Metabolic pathways involved in the treatment of XS volatile oil on OVX rats. (1) Phenylalanine, tyrosine, and tryptophan biosynthesis; (2) glycine, serine, and threonine metabolism; (3) aminoacyl-tRNA biosynthesis; (4) tyrosine metabolism; (5) tryptophan metabolism; (6) Pentose and glucuronate interconversions; (7) alanine, aspartate, and glutamate metabolism.

At the same time, metabolites were retained with *p* < 0.05 and fold change (FC) > 1.2 or <0.83 through the Metaboanalyst 5.0 database, and the filter rules were referred to other reports (Yuan et al., 2021). When the conditions of FC, *p*-value, and VIP-value were all satisfied, the metabolites were considered as differentially abundant. Metabolite changes induced by OVX and treatment of XS volatile oil are shown in Table 3 and Figure 9. We found the level of 21 metabolites significantly altered in the OVX group compared with the sham group, among which the levels of 2 metabolites were increased and those of 18 metabolites like l-tyrosine and l-tryptophan were reduced. XS volatile oil obviously recovered the levels of metabolites like l-tyrosine and l-tryptophan in OVX rats. Metabolic pathway analysis of these altered metabolites is shown in Figure 10. It showed that phenylalanine, tyrosine and tryptophan biosynthesis, tyrosine metabolism, and tryptophan metabolism were involved in the antidepressant effect of XS volatile oil in menopausal rats.

**TABLE 3:** Significantly changed metabolites between different groups.

No	Metabolites	OVX/sham				OVX-XSH/OVX			
		log2(FC)	−log10(*p*)	VIP	Trend	log2(FC)	−log10(*p*)	VIP	Trend
1	l-Cystine	−0.68	3.62	1.25	down	0.74	4.09	1.37	up
2	l-Lactic acid	−1.26	5.91	1.34	down	1.29	4.70	1.36	up
3	l-Alanine	−2.04	5.72	1.39	down	2.04	4.44	1.38	up
4	Epinephrine	0.46	3.50	1.18	up	−0.67	4.32	1.33	down
5	4-Hydroxybutyric acid	—	—	—	—	0.83	2.83	1.01	up
6	l-Valine	−1.46	4.60	1.37	down	1.49	3.94	1.37	up
7	Urea	−0.86	3.94	1.25	down	0.44	1.55	1.08	up
8	l-Proline	−1.61	4.39	1.36	down	1.63	3.75	1.34	up
9	l-Serine	−0.95	3.80	1.30	down	0.91	2.90	1.28	up
10	l-Threonine	−1.16	4.38	1.26	down	0.49	1.55	1.06	up
11	l-Asparagine	−0.89	3.25	1.22	down	—	—	—	—
12	d-Arabinose	0.86	2.47	1.02	up	−1.00	2.99	1.17	down
13	N-Acetylneuraminic acid	−2.32	5.83	1.38	down	2.47	4.70	1.37	up
14	l-Glutamine	−1.05	3.54	1.27	down	1.04	3.05	1.33	up
15	d-Galactose	−0.59	4.38	1.26	down	0.57	2.58	1.14	up
16	l-Lysine	−1.58	4.27	1.33	down	1.19	2.52	1.21	up
17	d-Glucose	−0.85	2.80	1.19	down	0.75	2.27	1.18	up
18	l-Tyrosine	−0.41	1.94	1.02	down	0.53	1.92	1.12	up
19	d-Xylulose	−1.24	3.30	1.24	down	1.24	2.99	1.26	up
20	Stearic acid	−0.29	3.27	1.14	down	—	—	—	—
21	L-Tryptophan	−1.14	5.21	1.32	down	0.78	2.25	1.08	up

## Discussion

Pathological studies have shown that menopausal syndrome mainly includes an imbalance of neurotransmitters, hormones, cytokines, and immune system (Stefanska et al., 2015), which increased the risk of depression. During menopause, estrogen levels were fluctuated, inducing depression and depressive-like behavior through interactions with neurotrophic factors and the serotonergic system. However, there is a lack of safe and effective drugs to treat menopausal depression.

TCM has been a great treasure in medical practice from Chinese history. The aromatic herbal pair of XS showed potential to relieve menopausal depression in previous studies (Ito et al., 2006; Ji et al., 2014a; Zhao et al., 2015), but its mechanism was poorly understood. With the development of modern science and technology, network pharmacology combined with metabolomics has been a promising strategy to illustrate the pharmacological mechanism of TCM, using which antidepressant actions of Xiaoyaosan and Huang-Lian Jie-Du decoction were illustrated (Liu et al., 2021; Qu et al., 2021). Thus, the present study aimed to study the antidepressant mechanism of XS volatile oil in menopausal rats by integrating network pharmacology with metabolomics.

In this study, a total of 64 constituents were identified from XS volatile oil. Perilla aldehyde (C11) from *Perilla frutescens* (L.) Britt., and cyperenone (C54), cyperene (C19), α-cyperone (C60), and dehydrofukinone (C45) from *Cyperus rotundus* L. were accounted for nearly 70% of all the detected chromatographic peaks. 38 active compounds like perilla aldehyde (C11), dehydrofukinone (C45), cyperenone (C54), and α-cyperone (C60) were further obtained by ADME screening, and they were predicted to be acting on 144 targets through the SwissTargetPrediction database. Among these targets, 42 targets were shared between compound-related and menopausal depression–related targets, indicating the possible antidepressant targets of XS volatile oil. Then the compound–target network of XS volatile oil against menopausal depression was constructed. According to the degree, betweenness centrality, and closeness centrality by topological analysis in this network, the top 10 ingredient nodes included dehydrofukinone (C45), cyperenone (C54), and α-cyperone (C60). Moreover, according to the previous studies, the sesquiterpenoid dehydrofukinone had sedative, anesthetic, and anticonvulsant effects through GABAergic or cortisol mechanisms (Garlet et al., 2016; Garlet et al., 2017). It was reported with anxiolytic-like effect in mice due to positive modulation of GABAA receptors and/or inhibition of neuronal calcium influx (Garlet et al., 2019). α-Cyperone was reported to exert antidepressant-like actions in a mice depression model which may be attributed to suppressing NLR family pyrin domain containing 3 (NLRP3) inflammasome (Xia et al., 2020).

Results of network pharmacology also suggested that targets such as *CYP19A1*, *CYP17A1*, *SLC6A4*, and *SLC6A3* were the crucial targets of XS volatile oil against menopausal depression ranked by degree, betweenness centrality, and closeness centrality. Go and KEGG enrichment analyses showed that XS volatile oil may exert its antidepressant effect by regulating monoamine transport and pathways of dopaminergic and serotonergic synapses. Moreover, *SLC6A4* was involved in the pathway of the serotonergic synapse, and *SLC6A3* was involved in the pathway of the dopaminergic synapse. Metabolomic analysis of the hippocampus in a rat model of CUMS-induced depression demonstrated that some altered metabolites were related to the serotonergic synapse, dopaminergic synapse, and glutamatergic synapse, which were involved in the pathology of depression (Gao et al., 2021). *SLC6A4* (a serotonin transporter gene) is one of the major determinants of serotonergic neurotransmission. It terminates neurotransmission by transporting serotonin from the synapse into the pre-synaptic nerve terminal. A comprehensive meta-analysis showed that serotonin transporter availability in depressed patients was reduced in key regions of the limbic system compared with healthy controls (Kambeitz and Howes, 2015). *SLC6A4* methylation was found to be positively correlated with stress and depression, and possessed the potential for the diagnosis and treatment of major depression (Okada et al., 2014; Park et al., 2019). *SLC6A3* (a dopamine transporter gene) affects the function of the dopamine nerve system. In geriatric patients with severe major depressive disorder, a low level of dopamine transporter binding was found in the region of the nucleus accumbens and putamen (Moriya et al., 2020). Alteration of dopamine transporter density in depressed patients with anhedonia showed reduced dopamine concentration in the synaptic cleft (Sarchiapone et al., 2006). *CYP19A1*-encoding aromatase is responsible for the key step in the synthesis of estradiol, which is widely expressed in the brain (Blakemore and Naftolin, 2016). Depletion of brain estrogen in middle-aged aromatase gene knockout (Ar^−/-^) mice increased the depressive-like behavior (Ma et al., 2020). All these results suggested that monoamine transport, and serotonergic and dopaminergic synapse pathways may be involved in the antidepressant action of XS volatile oil through network pharmacology analysis.

To verify the antidepressant effect of XS volatile oil, OVX combined with CUMS was established in rats to induce menopausal depression. As previously reported by others, OVX with CUMS resulted in several depressive-like behaviors in animals (Zhang et al., 2020; Zhou et al., 2020). In the present study, OVX induced a reduction of sucrose preference rate in SPT, a decrease of total traveled distance in OFT, and an increase of immobile time in FST, which could be reversed by XS volatile oil. Besides, XS volatile oil also upregulated the OVX-induced reduction of serum estradiol levels in rats. These results demonstrated the antidepressant-like effect of XS volatile oil.

In the following GC-MS–based non-targeted plasma metabolomic analysis, we found the level of 21 metabolites significantly altered in the OVX group compared with the sham group, among which the levels of two metabolites were increased and those of 18 metabolites like l-tyrosine and l-tryptophan were reduced. The results of reduced levels of l-tyrosine and l-tryptophan in this depressive animal model were in accordance with the results of CUMS depressive rats (Han et al., 2019), or the results of OVX-induced menopause (Lee et al., 2016). After XS volatile oil administration, l-tyrosine and l-tryptophan levels were upregulated in OVX rats. Metabolic pathway analysis was performed on these altered metabolites. It revealed that XS volatile oil may exert its antidepressant effect mainly by regulating phenylalanine, tyrosine and tryptophan biosynthesis, tyrosine metabolism, and tryptophan metabolism.

As we all know, amino acids have proven to be associated with the pathophysiological mechanism of depression (Chen et al., 2021). Tyrosine is one of the amino acids involved in dopamine biosynthesis. The dopamine system has been implicated in many different aspects of brain function, including locomotion, affect, and cognition. Many of the depressive symptoms, such as anhedonia and amotivation, have been more consistently associated with dysfunctions in the dopamine system (Grace, 2016). The decrease of dopamine release into the synapse caused altered expression of dopamine receptors within limbic structures, and that was observed in different depression models (Belujon and Grace, 2017). Similarly, tryptophan is a precursor highly essential for the brain regional synthesis of 5-hydroxytryptamine (5-HT, also known as serotonin). Patients with major depression had been confirmed with a deficit in circulation tryptophan, tryptophan uptake, serotonin synthesis and serotonergic neurotransmission (Ruddick et al., 2006; Gibson, 2018), and with decreased levels of brain serotonin and alterations in 5-HT receptors (Maes et al., 2009).

As reported by others, *Perilla frutescens* (L.) Britt. and *Cyperus rotundus* L. had the potential to protect against depression. For example, the essential oil of *Perilla frutescens* (L.) Britt effectively reversed the CUMS-induced reduction of brain-derived neurotrophic factors and improved the alterations of 5-HT concentrations in the hippocampus (Yi et al., 2013; Ji et al., 2014a). Perilla aldehyde, one of the active components in *Perilla frutescens* (L.) Britt, also exhibited an antidepressant-like effect in the CUMS-induced rat model of depression (Song et al., 2018). In lipopolysaccharide-induced depressive mice, pretreatment with perilla aldehyde reversed the decreased 5-HT levels in the prefrontal cortex, suggesting the antidepressant activity of perilla aldehyde might be related to the alteration of monoaminergic responses (Ji et al., 2014b). *Cyperus rotundus* L. was also frequently used in TCM prescription for treating depression in the clinic (Zhao et al., 2015). α-Cyperone, one of the active components in *Cyperus rotundus* L., also showed antidepressant action in a mice depression model (Xia et al., 2020).

The combination of network pharmacology and metabolomics has been an effective strategy to illustrate the antidepressive mechanism of TCM. For example, different efficacy groups of Xiaoyaosan showed synergistic antidepressant effects and contributed to the whole prescription against depression in CUMS rats (Liu et al., 2021). Huang-Lian Jie-Du decoction exhibited antidepressant effects by regulating *SLC6A4* and monoamine oxidase A (*MAOA*) in the tryptophan metabolism of CUMS depressive mice (Qu et al., 2021). In this study, integrated results of metabolomics and network pharmacology analysis indicated that XS volatile oil prevented depression through pathways including phenylalanine, tyrosine and tryptophan biosynthesis, tyrosine metabolism, and tryptophan metabolism, thus affecting serotonergic and dopaminergic synapses.

## Conclusion

In this study, through integrated methods of metabolomics and network pharmacology analysis, we found that XS volatile oil prevented the depressive-like behavior in OVX rats through regulating pathways including phenylalanine, tyrosine and tryptophan biosynthesis, tyrosine metabolism, and tryptophan metabolism to restore serotonergic and dopaminergic synapse. It preliminarily revealed the multi-compounds, multi-targets, and multi-mechanisms of XS volatile oil acting on menopausal depression. However, several limitations must be noted, such as the current results only exhibited metabolite changes in plasma, while changes in brain regions need investigation to further verify the antidepressant effect of XS volatile oil. Moreover, the verification of active components, targets, and pathways for XS volatile oil against menopausal depression could be complemented in the future study, such as detection of components in plasma, mRNA, and protein levels of *SLC6A4* and *SLC6A3*.

## Data Availability

The original data presented in the study are included in the article/Supplementary Material; further inquiries can be directed to the corresponding authors.
