# Supplementary figures and images for: Integrated Network Pharmacology and GC-MS–Based Metabolomics to Investigate the Effect of Xiang-Su Volatile Oil Against Menopausal Depression
Source: Front Pharmacol. 2021 Dec 2;12:765638. doi: 10.3389/fphar.2021.765638 (PMC8675254; doi:10.3389/fphar.2021.765638)

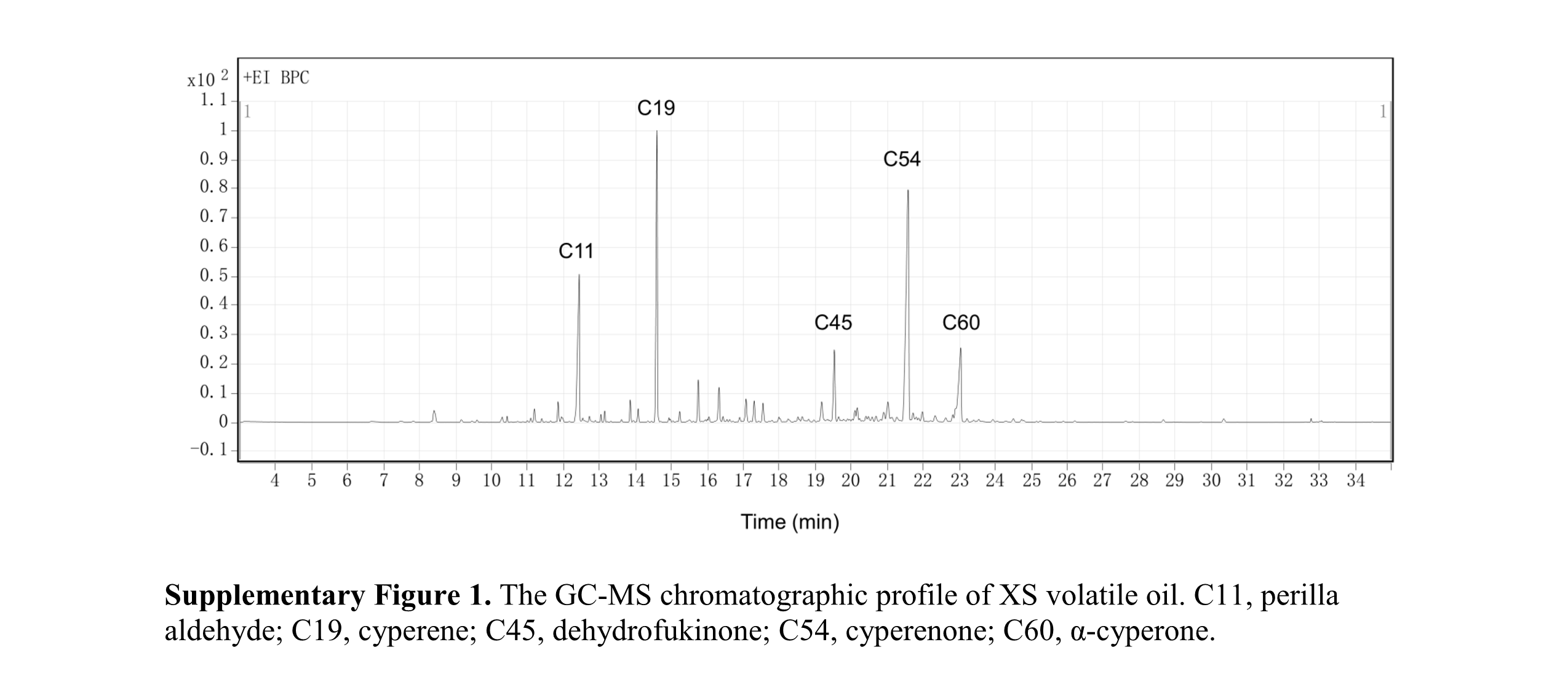

Supplement: Supplementary file 1 [file Image1.TIF]
